# Isolation and growth characterization of novel full length and deletion mutant human MERS-CoV strains from clinical specimens collected during 2015

**DOI:** 10.1099/jgv.0.001334

**Published:** 2019-10-08

**Authors:** Azaibi Tamin, Krista Queen, Clinton R. Paden, Xiaoyan Lu, Erica Andres, Senthilkumar K. Sakthivel, Yan Li, Ying Tao, Jing Zhang, Shifaq Kamili, Abdullah M. Assiri, Ali Alshareef, Taghreed A. Alaifan, Asmaa M. Altamimi, Hani Jokhdar, John T. Watson, Susan I. Gerber, Suxiang Tong, Natalie J. Thornburg

**Affiliations:** ^1^​ National Center for Immunization and Respiratory Diseases, Division of Viral Diseases, Gastroenteritis and Respiratory Viruses Laboratory Branch, Centers for Disease Control and Prevention (CDC), Atlanta, GA, USA; ^2^​ Batelle, Columbus, OH, USA; ^3^​ IHRC, Atlanta, GA, USA; ^4^​ Kingdom of Saudi Arabia Ministry of Health, Riyadh, Saudi Arabia

**Keywords:** Middle East respiratory syndrome human coronavirus, MERS-CoV, growth kinetics, clinical isolates, genomic sequences, coronavirus, phylogeny

## Abstract

Middle East respiratory syndrome (MERS) is a viral respiratory illness first reported in Saudi Arabia in September 2012 caused by the human coronavirus (CoV), MERS-CoV. Using full-genome sequencing and phylogenetic analysis, scientists have identified three clades and multiple lineages of MERS-CoV in humans and the zoonotic host, dromedary camels. In this study, we have characterized eight MERS-CoV isolates collected from patients in Saudi Arabia in 2015. We have performed full-genome sequencing on the viral isolates, and compared them to the corresponding clinical specimens. All isolates were clade B, lineages 4 and 5. Three of the isolates carry deletions located on three independent regions of the genome in the 5′UTR, ORF1a and ORF3. All novel MERS-CoV strains replicated efficiently in Vero and Huh7 cells. Viruses with deletions in the 5′UTR and ORF1a exhibited impaired viral release in Vero cells. These data emphasize the plasticity of the MERS-CoV genome during human infection.

## Introduction

Middle East respiratory syndrome coronavirus (MERS-CoV) is a Beta coronavirus that can cause respiratory illnesses that range in severity from asymptomatic to severe. Since the virus was identified in the Middle East in 2012, there have been over 2000 laboratory-confirmed cases with a mortality rate of approximately 35 % of reported cases (WHO). Over 80 % of cases have been reported in Saudi Arabia with all cases epidemiologically linked to the Arabian Peninsula. Dromedary camels have been identified as a primary animal reservoir, and human outbreaks have been postulated to be caused by spillover events [1]. As of September 2019, MERS-CoV continues to circulate.

MERS-CoV is an enveloped positive-strand RNA virus with a 30 kb genome that encodes 16 non-structural proteins within ORF1ab at the 5′ end and multiple structural and accessory proteins encoded at the 3′ end. To date, multiple recombinations and non-synonymous mutations have been identified in clinical specimens with the majority in Spike, Matrix, ORF1ab and ORF4b [2–5], but only eight deletion variants have been identified in viruses isolated from humans. All of the deletion variants have been in accessory and structural proteins. The identified deletions have been present in spike, ORF3a, ORF4, ORF5 and between ORF5 and E generating an ORF5E fusion protein [6–10]. Of note, the accessory proteins ORF4 and ORF5 are unique to Group 2C Beta coronaviruses and thus are absent in the other highly pathogenic Beta coronaviruses. None of the deletion variants identified in humans have been characterized *in vitro*, but the 414 nt deletion variant in ORF5 and E was identified in the index patient in China that had close contact with the index patient in South Korea in 2015 who was later deemed as a ‘super-spreader’ upon starting an outbreak that infected 186 people [7]. A 20 nt ORF5 deletion variant was identified from a fatal case occurred in 2015, linked to nosocomial transmission in Riyadh, Saudi Arabia [8]. Notably, deletion variants, with deletions in ORF3 and/or ORF4b have also been identified in specimens collected from dromedary camels in West and North Africa, which are phylogenetically distinct from viruses isolated from the Arabian Peninsula [11].

In this study, we have isolated eight MERS-CoV viruses from clinical specimens collected during 2015 in Saudi Arabia [2]. We sequenced the full genomes from all eight isolates and compared them to the full genome or spike gene from the matched clinic samples. Using these sequenced viral isolates, we also performed phylogenetic analysis and characterized the growth and viability of four viral isolates with both complete genomes and novel deletions in the highly conserved ORF1ab (nsp2), ORF3 and the 5′ UTR regions.

## Methods

### Clinical specimens

Specimens were collected as part of an epidemiologic investigation between January–June 2015 [2]. Respiratory specimens were collected and shipped to the Centers for Disease Control and Prevention. Portions of the specimens were used for RNA extraction and sequencing, which have been previously published [2]. Remaining respiratory specimens were used for virus isolation.

### Ethical approval

The use of human specimens to generate viral isolates was determined to be non-human subjects research by the National Center for Immunizations and Respiratory Diseases Human Research Protections Reviewer with the project ID P_2018_DVD_Thornburg_466.

### Cell culture, virus isolation, growth and titration

Vero and Huh-7 cells were obtained from ATCC and maintained in DMEM (Life Technologies, Grand Island, NY, USA) supplemented with 10 % heat-inactivated FBS (Hyclone, USA) and penicillin-streptomycin (PS) (Sigma-Aldrich, USA). Huh-7 cells were also supplemented with non-essential amino acids. MERS-CoV isolations were performed in sterile flat-bottom 96-well plates using Vero cells in a biosafety level 3 laboratory. Briefly, 50–100 µl respiratory specimens were serially diluted tenfold with DMEM and 100 ul of 3×10^5^ Vero cells ml^−^^1^ in suspension in DMEM/5 % FBS/PS. The specimens and cells were incubated at 37 °C/ 5 % CO_2_ and were monitored daily for signs of cytopathic effects (CPE). If wells showed CPE, half the well was used for passage, and half was used for RNA extraction and reverse transcription-polymerase chain reaction (RT-PCR) to confirm the presence of MERS-CoV. Isolates that were confirmed to be MERS-CoV were expanded and titred by TCID_50_ using crystal violet.

To characterize MERS-CoV growth kinetics, Vero or Huh-7 cells were plated in 24-well tissue-culture plates (Corning Costar), and cells were inoculated at an m.o.i. of 0.1 in 500 µl DMEM. After 30 min adsorption at 37 ˚C/5 % CO2, the monolayers were washed twice with PBS, and replaced with 1 ml of DMEM/5 % FBS/PS. The supernatants and cells were harvested every 12 h for 120 h. The titres of both supernatant and cell-associated viruses were determined by TCID_50_ in triplicate.

### Viral isolate sequencing and phylogenetic analysis

Overlapping primer pairs that span the entire MERS-CoV genome were developed and used in conjunction with the Fluidigm Access Array system [12]. The Fluidigm Access Array was used to set up and perform a throughput of 48 wells (15 samples with triplicate per sample) × 48 RT-PCRs according to the manufacturer’s protocol. Resulting amplicon pools from each sample were sheared from 800 to 1200 bp to 400–500 bp using a Covaris M220 sonicator. Sheared cDNA of each isolate was used to generate barcoded libraries for multiplexed sequencing using the NEB Next Ultra II DNA library prep kit (New England Biolabs). Sequencing was performed using an Illumina MiSeq instrument. Next-generation sequencing (NGS) data were analysed using a custom workflow in CLC Genomics Workbench 8.5. Trimmed reads were aligned to the reference Jordan-N3/2012 (KC776174.1). Consensus sequence was called based on regions that had 10× or greater coverage. The genome gaps after NGS sequence assembly were then filled by PCR followed by Sanger sequencing as described previously [12].

The genome nucleotide sequences from this study were first aligned in MAFFT v7.013 with published MERS-CoV sequences retrieved from GenBank. Phylogenetic trees were then inferred using the maximum-likelihood (ML) method using the available version 3.0 PhyML software assuming a general time-reversible (GTR) model with a discrete gamma distributed rate variation among sites (Gamma4) and a SPR tree swapping algorithm and visualized using mega version 6.

## Results

**Fig. 1.**
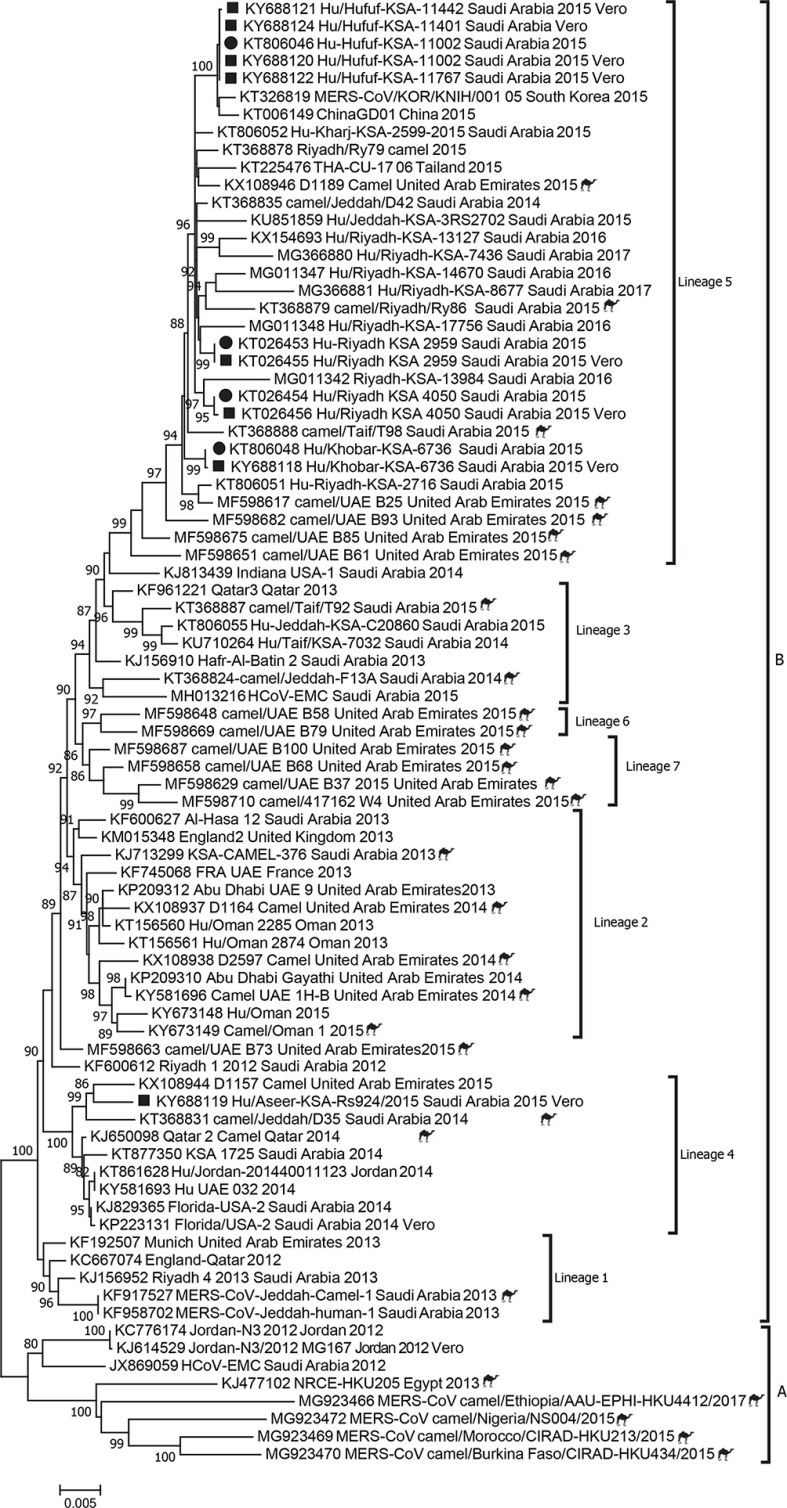
Phylogenetic analysis of MERS-CoV full genomes previously sequenced from humans and camels in comparison to novel clinical isolates (highlighted in grey).

Clinical specimens collected during an outbreak investigation had been previously characterized by qPCR and sequenced [2]. We used these clinical specimens for virus isolation. Several viruses were isolated from human respiratory clinical specimens including sputum, nasal swabs, nasopharyngeal or bronchoalveolar lavage in Vero cells. All viruses were isolated from specimens with Cts at or below 26. The full genome or spike gene of the novel isolates and the matched clinic samples were sequenced, phylogenetic analysis was performed, and sequences were compared to the sequences from the corresponding clinical specimens to identify potential tissue-culture adaptations. All viruses from this study were from clade B, lineages 4 and 5 (Table 1 and Fig. 1). The initial description of the sequences from these clinical specimens described these viral sequences as novel recombinant clade, however we have since adopted lineage language for clarity [2]. Of the eight isolates, five had full-length genomes, and three had genomic deletions. Four isolates with full-length genomes were isolated from clinical specimens in which the full genomes were sequenced. Six isolates had identical sequences in comparison to the available sequences (full genome or spike gene) of their matched clinical samples (Table 1), indicating no tissue-culture adaptations. The full-length isolate Hu/Khobar_KSA-6736_2015 had 1 nt non-synonymous change (F2242I) in ORF1ab, nsp3 in comparison to its source clinic specimen.

**Table 1.** Genomic characteristics MERS-CoV isolates

Isolate	Strain name	Clinical specimen accession	Isolate accession	Clade	Lineage	Genomic deletions
	Hu/Khobar_KSA_6736_2015	KT806048 (Full genome)	KY688118	B	5	Full length – 1 nt difference between clinical specimen and tissue-culture isolate
	Hu/Hofuf_KSA_11442_2015	KT806025 (Spike)	KY688121		5	Full length
	Hu/Hofuf_KSA_11767_2015	KT806031 (Spike)	KY688122		5	Full length
	Hu/Hofuf_KSA_11002_2015	KT806046 (Full genome)	KY688120		5	Full length
	Hu/Hofuf_KSA_11401_2015	KT806024 (Spike)	KY688124		5	Full length
	Hu/Riyadh_KSA_2959_2015	KT026453 (Full genome)	KT026455		5	Δ9 nt 5’ UTR, detected in clinical specimen and tissue-culture isolate
	Hu/Riyadh_KSA-4050_2015	KT026454 (Full genome)	KT026456		5	Δ3 nt ORF1a, Δ1AA NSP2
	Hu/Aseer_KSA_RS924_2015	KT805992 (Spike)	KY688119		4	Δ41 nt ORF3
Previously described	Hu/Florida/USA-2_Saudi Arabia_2014	n/a	KJ829365.1		1	Full length
	Hu/England-Qatar_2012	n/a	KC667074.1		4	Full length
	Hu/Jordan_N3_2012	n/a	KJ614529.1	A	A	Full length

Strain Hu/Riyadh_KSA_2959_2015 had a 9 nt deletion in the 5′ untranslated region (UTR), which was also present in the clinic sample, presumably resulting in no changes in viral protein sequences (Table 1). The isolate Hu/Riyadh_KSA-4050_2015 had a 3 nt deletion leading to a single amino acid deletion in NSP2 (I, at position 811) (Table 1) and a 1 nt synonymous change in the spike gene. The full genome of the matched clinical sequence was obtained, in which these mutations were absent, indicating potential tissue-culture adaptation (Table 1). Strain Hu/Aseer_KSA_RS924_2015 had a 41 nt deletion in ORF3 resulting in a frame shift (Table 1 and Fig. S1, available in the online version of this article). This deletion justified the distance of KY688119 by phylogenetic analysis (Fig. 1). Unfortunately, only the spike gene was sequenced from the clinical specimen due to very limited volume, so it is unclear if the deletion was generated during culturing. It remains a limitation of our comparison.

**Fig. 2.**
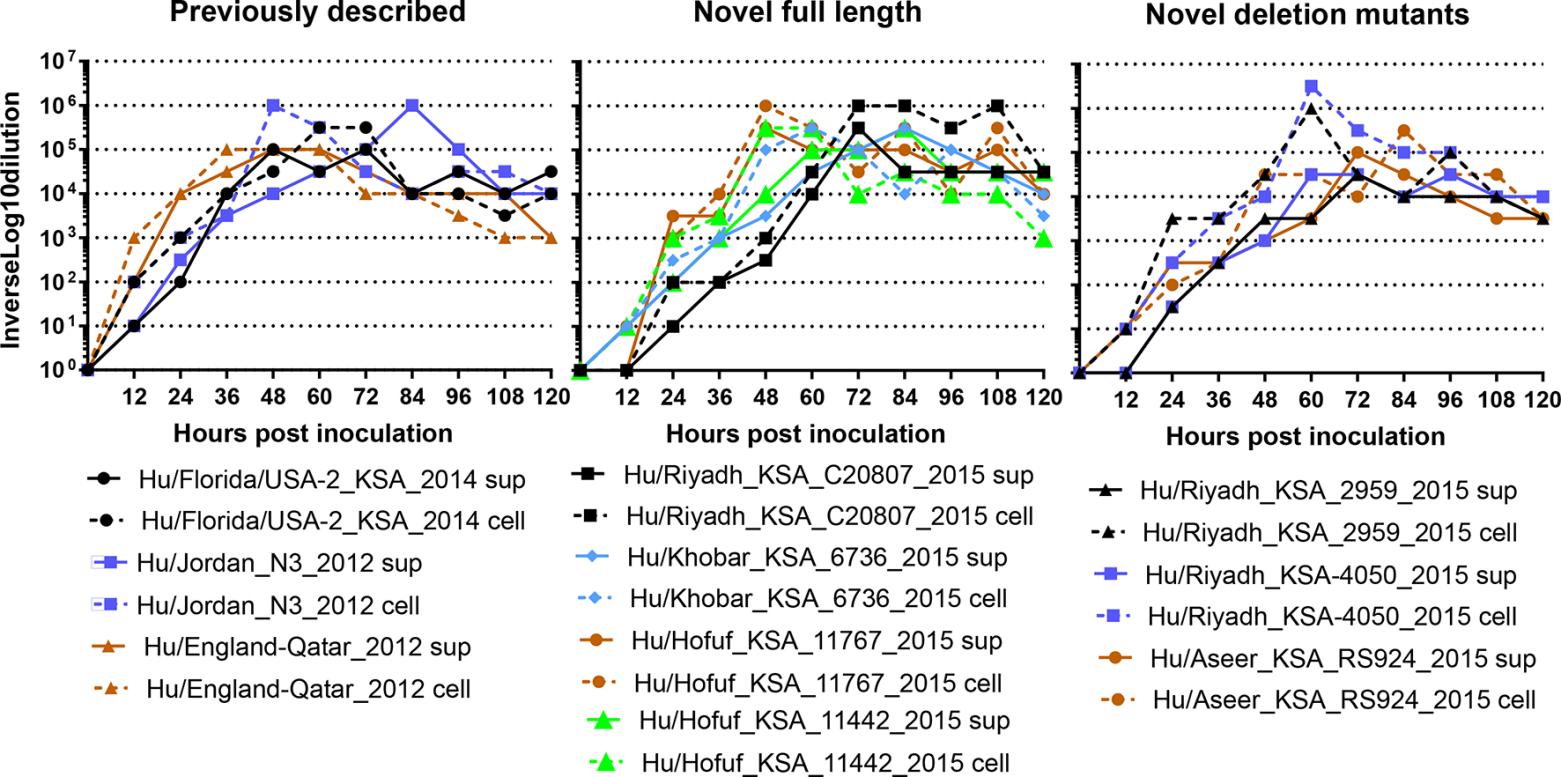
Multistep growth curve analysis in Vero cells. Vero-cell monolayers were inoculated at an m.o.i. of 0.1 with previously described, novel full length or novel deletion mutant MERS-CoV isolates. Cell and supernatants combined (a) or cell and supernatant separately (b) were collected every 12 h for 120 h. Timepoints were titred in Vero cells.

We performed growth-curve analysis on the selected virus isolates (three isolates with full genome and three isolates with deletions) from this study and three previously published viral isolates, novel full length and deletion mutant viruses in Vero cells to determine if genomic deletions corresponded with growth delays or inhibitions. Vero cells were inoculated with equivalent virus titres, and supernatants were collected every 12 h for 120 h and titrated by TCID50. Peak viral titres were observed between 48 and 96 h post inoculation, and did not differ between full-length isolates and deletion mutants (Fig. 2a). Peak titre of full-length viruses ranged from 10^5^ to 10^6^ TCID_50_ ml^−1^, whereas deletion mutants ranged from 5×10^4^−1×10^5^ TCID_50_ ml^−1^ (Fig. 2a), suggesting that these deletions may confer some growth inhibition, but not abrogate it.

In order to determine if the lower titres observed in the supernatants of Vero cells inoculated with deletion mutants represented lower viral replication or a budding deficiency, viruses were titred from Vero-cell lysates and supernatants after inoculation with full-length and deletion mutant viral isolates. In previously described and novel full-length viruses, the peak titre of each virus is approximately equal in cell-associated and supernatant fractions with a maximum of half a log difference (Fig. 2b). However, two of the three deletion mutant viruses exhibited higher titres in cell-associated fractions than supernatants (Fig. 2b). Hu/Riyadh_KSA_2959_2015 cell-associated titre peaked at 5×10^6^ TCID_50_ ml^−1^ with supernatant peaking at 5×10^4^ TCID_50_ ml^−1^. Similarly, Hu/Riyadh_KSA-4050_2015 cell-associated titre peaked at 1×10^6^ TCID_50_ ml^−1^ with supernatant peaking at 5×10^4^ TCID_50_ ml^−1^. The third deletion mutant virus Hu/Aseer_KSA_R2924_2015 exhibited similar titres in cell and supernatant fractions.

**Fig. 3.**
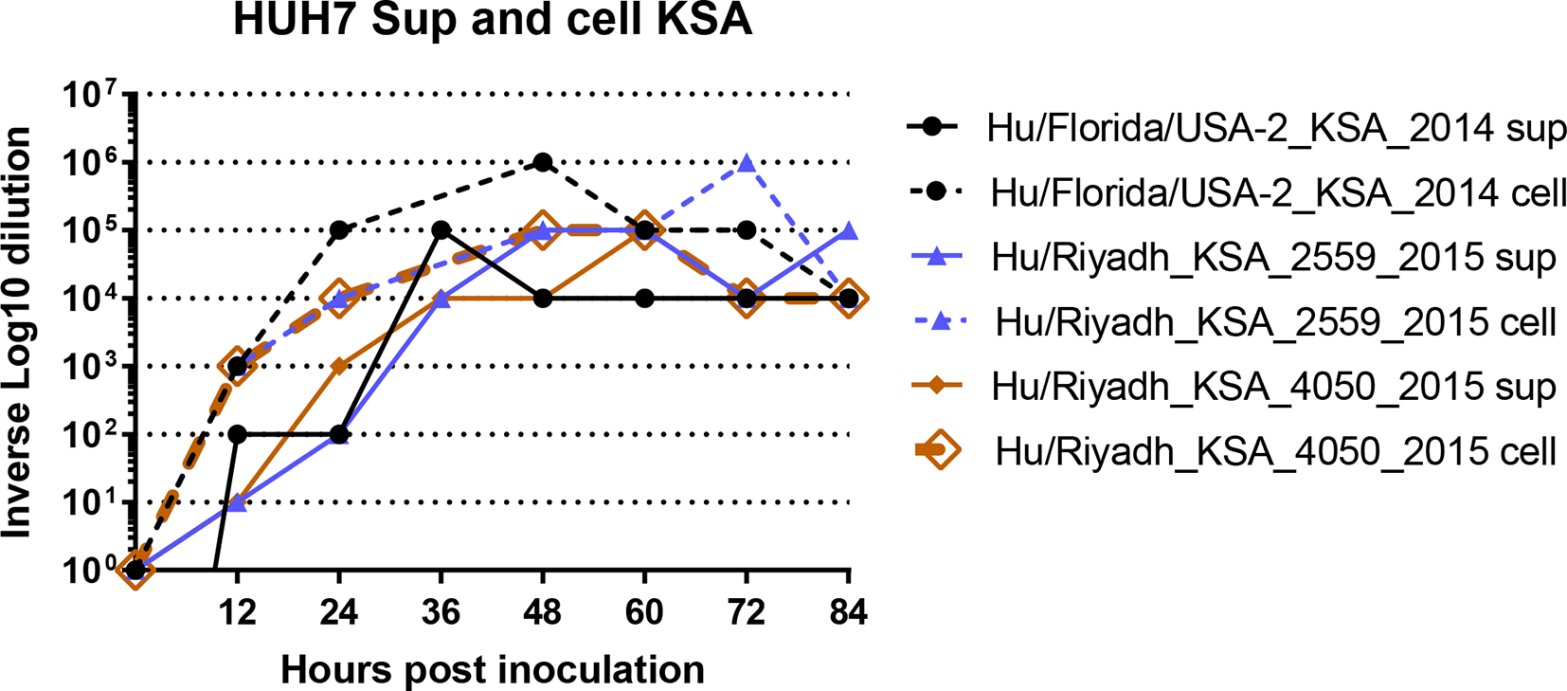
Multistep growth-curve analysis in HUH7 cells. HUH7 cell monolayers were inoculated at an m.o.i. of 0.1, with previously described Hu/Florida/USA-2_KSA_2014 or novel deletion mutants Hu/Riyadh_KSA_2014 or Hu/Riyadh_KSA_4050_2015, and cell and supernatants were collected separately every 12 h for 84 h. Cell and supernatant fractions were titred on Vero cells.

Genetically engineered viruses deleted in ORF 3, 4a, 4b and 5 exhibit efficient replication in Vero cells, but reduced replication in Calu3 cells [13]. Vero cells are known to be deficient in type I IFN responses, and MERS-CoV accessory proteins, ORF4a, ORF4b and ORF 5 have some IFN antagonist functions [14]. While none of our deletion mutants have deletions in ORF4a, ORF4b or ORF5, we wanted to test their abilities to replicate in cells with intact IFN systems. We tested the growth of full-length Hu/Florida/USA-2_KSA_2014, Hu/Riyadh_KSA_2959_2015 and Hu/Riyadh_KSA_4050_2015 in Huh7 cells, which have an intact IFN system. Neither deletion mutant exhibited delayed growth or reduced titres (Fig. 3), indicating they have an intact ability to antagonize the IFN system.

## Discussion

In this manuscript, we describe eight MERS-CoV isolates collected from patients in 2015 in Saudi Arabia. Of those isolates, all were identified to be in clade B, five had full-length genomes, and three exhibited genomic deletions. This work is the first example of *in vitro* characterization of human clinical isolates with genomic deletions. These deletions were located in the 5′ UTR, ORF1a in NSP2, and ORF3. Growth-curve analysis indicated the viruses still grew efficiently in cell culture, however Hu/Riyadh_KSA_2959_2015 and Hu/Riyadh_KSA-4050_2015 exhibited higher cell-associated viral titres than titres in supernatant, indicating they may have a deficiency in viral budding or release.

Other MER-CoVs have been isolated from specimens collected from dromedary camels in North and West Africa with deletions in ORF3 and/or ORF4b [11]. The location and size of the deletions varied, however many resulted in frame shifts or premature stop-codons. Notably, these viruses were of a distinct clade (clade C1), but still had deletions in a similar region to our isolates from clade B human viruses. The clade C camel MERS viruses replicated less efficiently in Calu-3 cells than the human MERS-CoV isolate, EMC. These deletion mutants exhibited increased type I and III IFN responses with increased mRNA expression of IFN-β and IFN-γ1. When the deletion was engineered into a human strain of MERS-CoV (EMC), the viruses also exhibited higher type I and III, but did not exhibit growth inhibition suggesting the deletion is not solely responsible for growth inhibition. These data are consistent with the growth characteristics of the viruses described in our study.

It is clear from the ability of these novel MERS isolates with deletions, that none of the deletions dramatically affected the ability of the viruses to replicate in cell culture. This is not surprising given that two of the deletions are located in accessory proteins, and one is outside of a coding region. There may be compensating mutations elsewhere in the genome, however we have not yet been able to identify any, despite full-genome analysis.

Hu/Aseer_KSA_RS924_2015 exhibited a 41 nt deletion in ORF3 (Fig. S1). The function of ORF3 is unknown, but the protein is conserved across coronaviruses. Other groups have generated coronavirus mutants lacking ORF3, 4a, 4b and 5 that also replicate efficiently in cell culture [13, 15, 16]. Replication of an engineered quadruple deletion mutant was attenuated Calu3 cells but not in IFN-defective Vero cells, suggesting host response may affect the ability of these deletion mutants to grow [13, 15, 16]. This deletion mutant also induced elevated IFNβ and IFNγ in cell culture, and exhibited reduced replication in mice suggesting a potential role in pathogenesis [13]. Hu/Aseer_KSA_RS924_2015 was isolated from an ill hospitalized patient without comorbidities who was eventually discharged to home. Despite the large deletion and frame shift in ORF3, Hu/Aseer_KSA_RS924_2015 still retained the ability to cause illness. Unlike the engineered mutant lacking ORF3, 4a, 4b and 5 generated by reverse genetics, Hu/Aseer_KSA_RS924_2015 still retained most of ORFs 4a, 4b and 5, which may be stronger contributors to pathogenesis than ORF3.

The deletion in ORF1ab in Hu/Riyadh_KSA-4050_2015 maps to nsp2. SARS-CoV nsp2 is dispensable for replication, however it has been found to bind host proteins, prohibitin 1 and prohibitin 2 [17]. These direct protein–protein interactions have been hypothesized to alter the host environment. We have not yet examined similar functions with Hu/Riyadh_KSA-4050_2015, but because this deletion is absent in the matched clinical specimen, it may indicate a tissue-culture adaptation.

The deletion mutant, Hu/Riyadh_KSA_2959_2015, exhibited a 9 nt deletion in the 5′ UTR. The 5′ UTR is thought to play a role in the discontinuous synthesis of subgenomic RNAs as well as bind several host proteins [18]. Limited research has been done on the roles of group-specific accessory proteins or regions such as the 5′ UTR in replication, virulence and pathogenicity.

Efficient replication of viruses with genomic deletions in cell culture do not eliminate the possibility that the gene products may play important roles *in vivo*. All of these viruses were isolated from ill patients. Hu/Riyadh_KSA_2959_2015 (Δ9 nt 5′ UTR) and Hu/Aseer_KSA_RS924_2015 (Δ41 nt ORF3) were isolated from patients who eventually died, and Hu/Riyadh_KSA-4050_2015 (Δ3nt ORF1a) was isolated from a hospitalized patient who was discharged to home. We confirmed the deletion of the 5′ UTR in the matched clinical specimen. The patient infected with Hu/Riyadh_KSA-4050_2015 (Δ3nt ORF1a) died during the course of illness confirming that the virus mutant retained its pathogenicity for humans. The 3 nt deletion in ORF1a in Hu/Riyadh_KSA-4050_2015 was absent in the matched clinical specimen suggesting that only full-genome viruses in the sample were able to grow in tissue culture. Additionally, we were unable to confirm that the ORF3 deletion in Hu/Aseer_KSA_RS924_2015 is also present in the clinical specimen because we were only able to recover the spike sequence from the clinical specimen due to limited volume. It is a limitation of the study. The patient from which Hu/Aseer_KSA_RS924_2015 (ΔORF3) was isolated died during the course of the illnesses. By isolating and characterizing MERS-CoV deletion variants detected in ill patients, we have confirmed that these viruses are viable and are not simply an artifact of directly sequencing clinical specimens. We have not yet determined if these deletions render less pathogenic viruses, however pathogenesis studies in animal models of these isolates are ongoing.

The findings and conclusions in this report are those of the authors and do not necessarily represent the official position of the Centers for Disease Control and Prevention. Names of specific vendors, manufacturers or products are included for public health and informational purposes; inclusion does not imply endorsement of the vendors, manufacturers or products by the Centers for Disease Control and Prevention or the US Department of Health and Human Services.

## Supplementary Data

Supplementary File 1
