# Supplementary material for: Isolation and growth characterization of novel full length and deletion mutant human MERS-CoV strains from clinical specimens collected during 2015
Source: J Gen Virol. 2019 Oct 8;100(11):1523–9. doi: 10.1099/jgv.0.001334 (PMC7079693; doi:10.1099/jgv.0.001334)
Supplement: Supplementary File 1 [file jgv-100-1523-s001.pdf]

```
Hu/Aseer_KSA_RS924_2015   25532 ATGAGAGTTCAAAGACCACCCACTCTCTTGTTAGTGTTCTCACTCTCTCTTTTGGTCACT 25591
Hu/England-Qatar_2012     25532 AUGAGAGUUCAAAGACCACCCACUCUCUUGUUAGUGUUCUCACUCUCUCUUUUGGUCACU 25591

Hu/Aseer_KSA_RS924_2015   25592 GCATTTTCAAAACCTCTCTATGTACCTGAGCATTGTCAGAATTATTCTGGTTGCATGCTT 25651
Hu/England-Qatar_2012     25592 GCAUUUUCAAAACCUCUCUAUGUACCUGAGCAUUGUCAGAAUUAUUCUGGUUGCAUGCUU 25651

Hu/Aseer_KSA_RS924_2015   25652 AGGGCTTGTATTAAAACTGCCCAAGCTGATACAGCTGGTCTTTATACAAATTTTCGAATT 25711
Hu/England-Qatar_2012     25652 AGGGCUUGUAUUAAAACUGCCCAAGCUGAUACAGCUGGUCUUUAUACAAAUUUUCGAAUU 25711

Hu/Aseer_KSA_RS924_2015   25712 GACGTCCCATCTGCAGAATCAACTGGTACTCAATCAGTTTCTGTCGATCGTGAGTCAACT 25771
Hu/England-Qatar_2012     25712 GACGUCCCAUCUGCAGAAUCAACUGGUACUCAAUCAGUUUCUGUCGAUCGUGAGUCAACU 25771

Hu/Aseer_KSA_RS924_2015   25772 TCAACTCATGATGGTCCT-----------------------------------------C 25790
Hu/England-Qatar_2012     25772 UCAACUCAUGAUGGUCCUACCGAACAUGUUACUAGUGUGAAUCUUUUUGACGUUGGUUAC 25831

Hu/Aseer_KSA_RS924_2015   25791 TCAGTTAATTAA 25802
Hu/England-Qatar_2012     25832 UCAGUUAAUUAA 25843
```

Supplementary figure 1. Nucleotide alignment of ORF3 Hu/Aseer_KSA_RS924_2015 and Hu/England-Qatar_2012
